# A High-Strain-Rate Superplasticity of the Al-Mg-Si-Zr-Sc Alloy with Ni Addition

**DOI:** 10.3390/ma14082028

**Published:** 2021-04-17

**Authors:** Andrey Mochugovskiy, Anton Kotov, Majid Esmaeili Ghayoumabadi, Olga Yakovtseva, Anastasia Mikhaylovskaya

**Affiliations:** Department of Physical Metallurgy of Non-Ferrous Metals, National University of Sciences and Technology ‘‘MISIS”, 4 Leninskiy ave. 4, 119049 Moscow, Russia; mochugovskiy.ag@misis.ru (A.M.); kotov@misis.ru (A.K.); ilieamse_dijam@yahoo.com (M.E.G.); yakovtseva.oa@misis.ru (O.Y.)

**Keywords:** superplasticity, aluminum alloys, transition metals, dispersoids, particle-stimulated nucleation

## Abstract

The current study analyzed the effect of Ni content on the microstructure and superplastic deformation behavior of the Al-Mg-Si-Cu-based alloy doped with small additions of Sc and Zr. The superplasticity was observed in the studied alloys due to a bimodal particle size distribution. The coarse particles of eutectic origin Al_3_Ni and Mg_2_Si phases with a total volume fraction of 4.0–8.0% and a mean size of 1.4–1.6 µm provided the particles with a stimulated nucleation effect. The L1_2_– structured nanoscale dispersoids of Sc- and Zr-bearing phase inhibited recrystallization and grain growth due to a strong Zener pinning effect. The positive effect of Ni on the superplasticity was revealed and confirmed by a high-temperature tensile test in a wide strain rate and temperature limits. In the alloy with 4 wt.% Ni, the elongation-to-failure of 350–520% was observed at 460 °C, in a strain rate range of 2 × 10^−3^–5 × 10^−2^ s^−1^.

## 1. Introduction

AA6XXX-type (Al-Mg-Si-based) alloys are widely applied for engineering applications [1,2], owing to their low density, high corrosion resistance, and increased mechanical properties at room temperature. The Al-Mg-Si-based alloys belong to the heat treatable group due to a significant strengthening effect, provided by β′, β″-phases (metastable modifications of the Mg_2_Si-phase), usually form during the ageing process [3,4,5,6]. Numerous commercial 6XXX-type alloys contain Cu that improves strength owing to the formation of the metastable Q-(Al,Mg,Si,Cu)-type phase precipitates [3,7,8,9]. Due to the promising mechanical characteristics at room temperature and a low critical cooling rate for solid solution treatment, the Al-Mg-Si-based alloys are attractive for production of the complex-shaped parts by superplastic forming (SPF) technology. The superplasticity of Al-Mg-Si based alloys is poorly studied. The main problems of Al-Mg-Si based alloys are the difficulty of the grain refinement and the significant grain growth that caused the low strain rate sensitivity and weak superplasticity [10]. A comparatively low alloyed solid solution [11,12] is prone to the grain growth during heating up to the superplastic deformation temperature and during superplastic deformation that decrease an elongation-to-failure [10], and led to the “orange peel” effect on the post-forming surface [13]. Some progresses in the field of superplasticity for the Al-Mg-Si based alloys have been made by Troeger and Stark [14,15]. The authors applied Rockwell-type technology, including heterogenization annealing [16] for the AA6061 alloy, to involve the particle stimulated nucleation (PSN) effect. The thermomechanical treatment provides fine-grained structure with a grain size of about 9 µm and superplasticity with the elongation-to-failure of 375% at a strain rate of 5 × 10^−4^ s^−1^. Moreover, a low strain rate is a cause of low SPF productivity. A significant progress in the grain refinement of Al-Mg-Si bases alloys was attained due to severe plastic deformation (SPD) techniques. A mean grain size of 0.24–4.5 µm, and superplasticity in a wide strain rate range of 1 × 10^−4^–5 × 10^−2^ s^−1^ were achieved for the 6061 and 6063 alloys processed by equal-channel angular pressing and accumulative roll bonding, while the elongation-to-failure was 200−270% [17,18,19]. Nevertheless, low elongation, which is the result of a significant grain growth, limits complex shaped parts production by SPF method. In addition, most of the SPD techniques cannot be conveniently applied for mass production, due to financial and technical aspects. 

The modification of the alloys’ composition is an effective way to provide a thermally stable fine-grained structure and a good superplasticity for various alloys [20]. In this case, the grain-refinement approach is based on the bimodal particle size distribution that can be arranged after a simple thermomechanical treatment [20]. Coarse particles provide grain refinement during recrystallization via particle-stimulated nucleation (PSN) effect [16,21,22]. Such coarse particles may precipitate during heterogenization treatment from a solid solution supersaturated by Mg, Zn, Si, Cu [14,16,23], and precipitate during solidification due to alloying with eutectic forming elements, such as Ni, Fe, Mn, Ce [24,25,26,27]. Nickel is a promising alloying element for increasing the superplasticity of Al-based alloys [24,26,27,28,29,30]. The homogeneous distribution of the coarse Al_3_Ni particles with a near-spherical morphology is formed in the alloys’ sheets during a simple thermomechanical treatment [26,27,29]. Fine dispersoids are formed due to alloying with rare-earth (RE) and transition metals (TM), such as Zr, Sc, Mn, Cr, etc. Nanoscale dispersoids are precipitated during thermomechanical treatment from a supersaturated by TM/RE aluminum based solid solution [31,32,33,34]. Dispersoids cause Zener pinning effect and stabilize grain size [35,36,37,38]. The joint addition of Sc and Zr is the most effective dispersoids-forming combination. Sc and Zr provide the formation of a high density core-shell L1_2_-strucured Al_3_(Sc,Zr) dispersoids [39,40,41]. These dispersoids result in strong Zener pining effect and inhibit grain growth, and, in addition, increase tensile properties at room temperature [42,43,44,45,46,47,48]. In novel alloys, the additions of Sc, as an expensive element, are reduced to 0.1 wt.%, while that of Zr is retained in a higher amount, 0.2 wt.% [32,49,50]. The bimodal distribution of the particles in the alloy’s structure provides a high strain rate superplasticity with strain rate sensitivity coefficient *m* above 0.3 for the AA5XXX- [24,30,51], AA7XXX- [29,30,42,52] and AA2XXX-types [27,34] alloys containing eutectic- and dispersoid-forming Fe [24], Ni [24,27,29,52], Ce [25,27,53], Y [34], Er [51], Mn [54,55], Cr [55], Zr [30,56,57], Sc [58,59,60,61]. The influence of the eutectic-forming and dispersoid-forming elements on the grain structure and superplastic deformation behavior of Al-Mg-Si type alloys is poorly studied. The current work focuses on the analysis of the microstructural evolution and superplastic behavior of a novel Al-Mg-Si-based alloy with different content of eutectic forming Ni and small additives of dispersoid forming Sc and Zr.

## 2. Materials and Methods

A novel Al-Mg-Si-Cu-Ni-Zr-Sc alloy was studied. The concentrations of the alloying elements are shown in Table 1.

The alloys were prepared in a 20 kW induction furnace (Interselt, Saint-Petersburg, Russia) using graphite-fireclay crucibles (Lugaabrasiv, Luga, Russia). For the preparation of the alloys, the following pure metals and “master alloys” (UC Rusal, Moscow, Russia) were used: Al (99.99 wt.%), Mg (99.96 wt.%), Al-5 wt.% Zr, Al-2 wt.% Sc, Al-12 wt.% Si, Al-53.5 wt.% Cu, and Al-20 wt.% Ni. The temperature was controlled by a chromel-alumel thermocouple. The temperature of the melt before casting was 830 ± 10 °C. The casting was performed using water-cooled copper mold with a size of 100 × 40 × 20 mm^3^, providing the casting cooling rate of approximately 15 K/s.

The heat treatment of as-cast material plays the most important role for the dispersoid strengthening and grain boundary pinning effect. The high density of fine L1_2_-strucutred Sc- and Zr-bearing dispersoids is observed after low temperature annealing [43,62,63,64,65] and two-step annealing regimes [43,56,63,65,66,67]. The homogenization annealing for the studied alloys was performed in two steps, with the first low-temperature step at 350 °C for 8 h and the second high-temperature step at 480 °C for 3 h to provide precipitation of the L1_2_ dispersoids and fragmentation and spheroidization of the Al_3_Ni particles [29,52]. The as-homogenized alloy was subjected to a hot rolling (Rolling mill V-3P, GMT, Saint-Petersburg, Russia), with 85% total reduction and subsequent cold rolling (Rolling mill V-3P, GMT, Saint-Petersburg, Russia),), with 65% total reduction to prepare sheets with a thickness of 1 mm. The hot rolling was performed at 400 ± 20 °C. The hot and cold rolling were performed in 6 and 10 passes, respectively.

The phase composition of the alloys in the as-cast state was controlled by X-ray diffraction (XRD) analysis, using Bruker D8 Advance diffractometer with Cu-K_α_ radiation (Bruker Corporation, Billerica, MA, USA).

The microstructural evolution was studied by electron scanning microscopy (SEM) and optical microscopy (OM). A Tescan-VEGA3 LMH electron microscope with a tungsten filament cathode (Tescan Brno s.r.o., Kohoutovice, Czech Republic) and an Oxford Instruments Advanced AZtecEnergy energy dispersive X-ray microanalysis system (EDS) (Oxford Instruments plc, Abingdon, UK) was used in the current study. Backscattered electron imaging (BSE) was performed with a 15 mm work distance and acceleration voltage of 20 kV. The grain structure of samples before the superplastic deformation was analyzed under a Carl Zeiss Axiovert 200M optical microscope (Carl Zeiss, Oberkochen, Germany) in a polarized light. The samples for OM and SEM analysis were prepared by mechanical grinding with subsequent polishing using the colloidal silica suspension Struers OP-U( Struers APS, Ballerup, Denmark). The samples for OM were subjected to anodic oxidation in a 10% water solution of the H_3_BO_4_ saturated in the HF.

The transmission electron microscopy (TEM) analysis was carried out using a JEOL JEM 2100 microscope (JEOL, Tokyo, Japan). The disc-shaped samples of 3 mm diameter and 0.18–0.25 mm thickness were used for TEM analysis. The samples were preliminarily subjected to a twin-jet electropolishing in a Struers TenuPol-6 system (Struers APS, Ballerup, Denmark) at −25 ± 5 °C using 30% HNO_3_ methanol solution (Struers APS, Ballerup, Denmark).

The tensile test at elevated temperatures was performed using a Walter-Bai LFM 100 universal testing machine (Walter + Bai AG, Löhningen, Switzerland) in a temperature range of 440–500 °C and a constant strain rate range of 2 × 10^−3^–5 × 10^−2^ s^−1^. Three samples with a gauge part size of 14 × 6 × 1 mm^3^ were used for each testing condition. The largest dimension of the gauge part was parallel to the rolling direction. The strain rate was maintained constant due to controlled increasing travers speed during the test. The strain rate sensitivity *m* coefficient values were calculated as a first derivative of the logarithmically transformed Backofen equation for a strain of 50%.

(1) $$m=\partial \ln\sigma /\partial \ln\dot{\varepsilon }$$

where *σ* is flow stress and $\dot{\varepsilon }$ is strain rate.

## 3. Results

### 3.1. Phase Analysis

**Figure 1:**
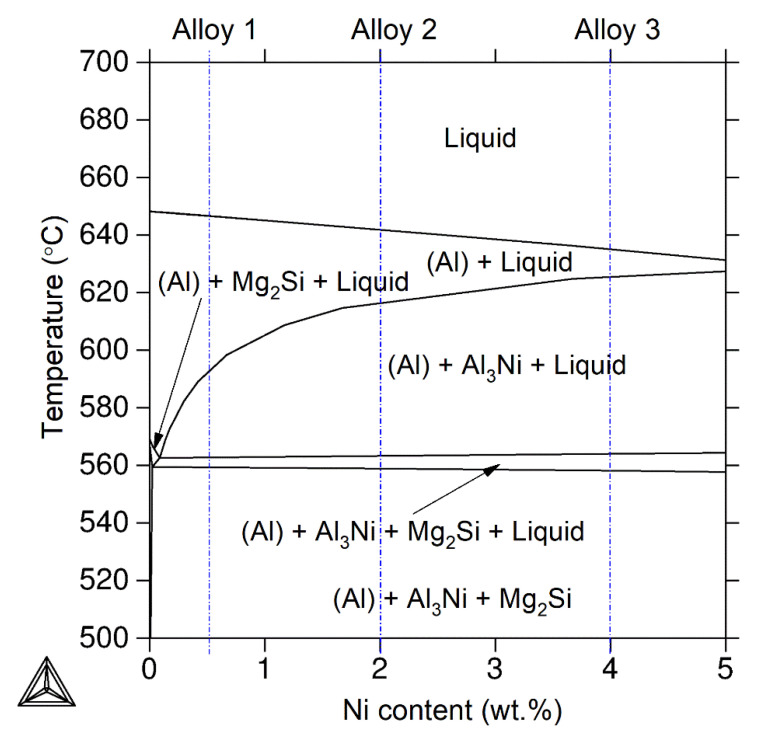
The polythermal section of the equilibrium Al-1.2%Mg-0.7%Si-1.0%Cu-var.%Ni phase diagram; blue vertical lines show the studied alloys compositions.

Figure 1 shows the polythermal section of the Al-Mg-Si-Cu-Ni equilibrium phase diagram for the Ni concentration of below 5 wt.% calculated in a ThermoCalc (ThermoCalc-database TTAl5, Thermo-Calc Software AB, Stockholm, Sweden). The studied alloys are marked in the diagram by the blue vertical lines. The following phases were sequentially solidified for the alloys studied in the equilibrium state: the aluminum-based solid solution (Al), the Al_3_Ni and Mg_2_Si phases.

### 3.2. XRD Analysis

**Figure 2:**
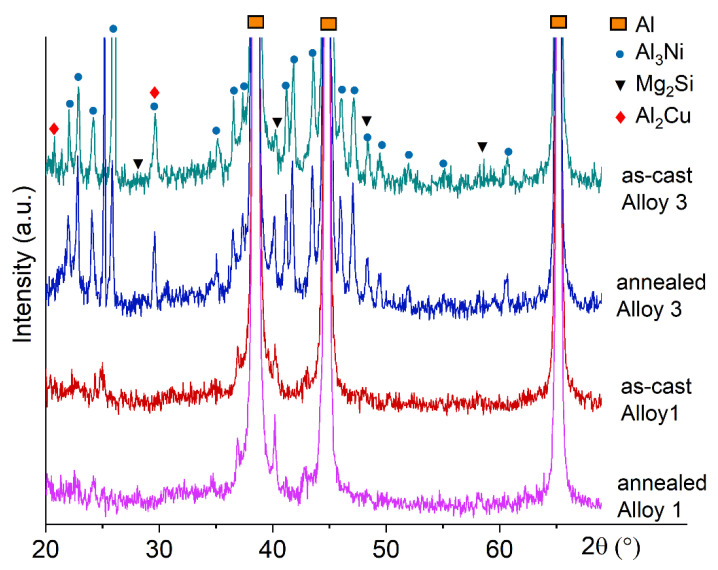
XRD spectrums for the alloys 1 (Al-1.2Mg-0.7Si-1.0Cu-0.5Ni) and 3 (Al-1.2Mg-0.7Si-1.0Cu-4Ni) in as-cast and annealed states.

The XRD analysis was performed to determine the phase composition of Alloys 1 and 3 (Figure 2). The diffractions peaks corresponding to the (Al), Al_3_Ni, Al_2_Cu, and Mg_2_Si phases were detected in as-cast state. The (Al), Al_3_Ni, and Mg_2_Si phases were found in as-annealed state.

### 3.3. SEM Analysis

**Figure 3:**
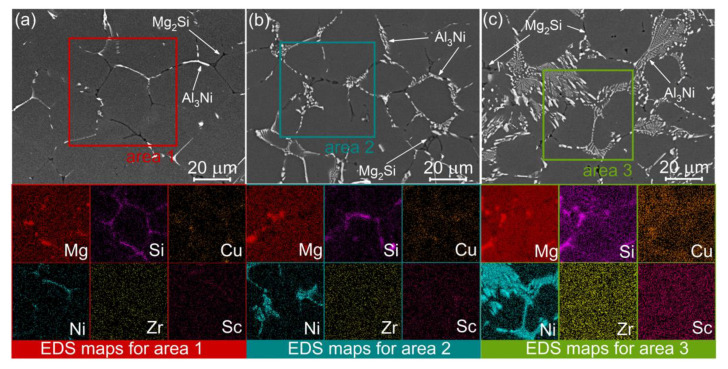
BSE images corresponding to SEM-EDS maps for the (**a**) alloy 1 (Al-1.2Mg-0.7Si-1.0Cu-0.5Ni), (**b**) alloy 2 (Al-1.2Mg-0.7Si-1.0Cu-2Ni), and (**c**) the alloy 3 (Al-1.2Mg-0.7Si-1.0Cu-4Ni) in as-cast state.

Figure 3 shows the BSE images for the studied alloys in the as-cast state. The dominant microstructural feature was the dendrites of the Al-based solid solution. The two types of crystallization-origin phases surrounded the dendrite cells of Al solid solution in the studied alloys; predominantly, the Ni-rich bright phase and the dark Mg- and Si-bearing phase (Mg_2_Si). The volume fraction of Ni-bearing phase expectedly increased with increasing Ni content. The colonies of fine lamellar eutectic were observed in alloys 2 and 3 (Figure 3b,c), whereas the bright phase formed the planes on the periphery of dendrite cells in the alloy 1 with low Ni content (Figure 3a). The volume fraction of the Ni-rich particles was 2.8 ± 0.4%, 4.8 ± 0.3%, and 8.2 ± 0.4% for the alloys 1, 2, and 3, respectively. The Mg_2_Si phase appeared predominantly on the periphery of dendrite cells and its volume fraction of 1.2 ± 0.3% was similar for the studied alloys. An incomplete superposition of Mg-rich and Si-rich zones in the EDS-maps suggested the presence of a small fraction of (Si)-phase in as-cast state. The EDS analysis demonstrated the Cu segregations on the periphery of the dendrite cells. The Sc and Zr-bearing phases of solidification origin were unobserved and the EDS maps for Sc and Zr exhibited uniform elemental distribution for the alloys.

**Figure 4:**
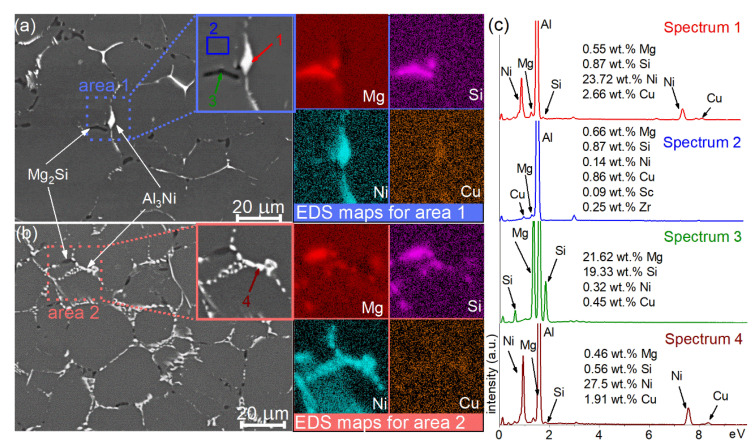
SEM images for the (**a**) alloy 1 (Al-1.2Mg-0.7Si-1.0Cu-0.5Ni) and (**b**) alloy 2 (Al-1.2Mg-0.7Si-1.0Cu-2Ni) after the two-step homogenization and corresponded to EDS elemental distribution maps captured from the area 1 in (**a**) and area 2 in (**b**); (**c**) the EDS spectrums corresponded to the area marked-up in insert in (**a**,**b**).

**Figure 5:**
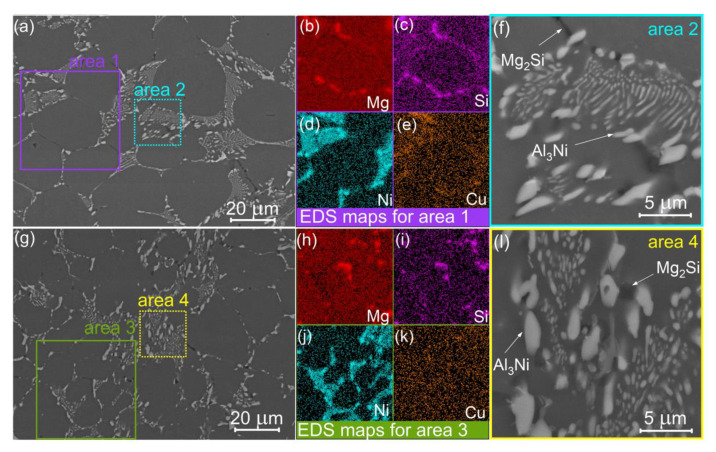
SEM images for the alloy 3 (Al-1.2Mg-0.7Si-1.0Cu-4Ni) after (**a**–**f**) one step homogenization at 350 °C, 8 h and (**g**–**l**) two-step with the first step at 350 °C, 8 h and second step at 480 °C, 3 h; (**b**–**e**) and (**h**–**k**) are the EDS maps captured from the are marked up with red square in (**a**) and green square in (**g**), respectively; (**f**) and (**l**) are the magnified areas marked up with blue square in (**a**) and yellow square in (**g**), respectively.

SEM micrographs for as-homogenized samples are presented for the alloys 1 and 2 in Figure 4 and for the alloy 3 in Figure 5. The first annealing step at 350 °C, 8 h, did not initiate the significant changes in the as-cast microstructure for the studied alloys (Figure 5a–f). The Al_3_Ni phase retained lamellar morphology, and a partial fragmentation of Mg- and Si-bearing phase was observed (Figure 5f). The chemical composition of the phases was studied in detail after the second step of homogenization at 480 °C. According to the SEM-EDS data, the bright Ni-bearing phase contained Cu (Figure 4c).

The EDS analysis revealed that the bright phase contained 23.7 wt.%Ni, 2.7 wt.% Cu for the alloy 1 (Figure 4 spectrum 1), and 7.5 wt.% Ni, 1.9 wt.% Cu for the alloy 2 (Figure 4 spectrum 4). The dark phase contained 21.6 wt.% Mg and 19.3 wt.% Si (Figure 4 spectrum 3), whereas the total concentration of the other elements was less than 1 wt.%. The analysis of Al solid solution revealed 0.7% Mg, 0.9% Si, and 0.9% Cu (Figure 4 spectrum 2). For the alloy 3, the Cu-distribution became more homogeneous after high-temperature annealing. However, a slight increase of the Cu signal was noticed near the Ni-rich zones.

The second step of homogenization annealing resulted in the fragmentation and spheroidization of the Mg_2_Si phase for the studied alloys (Figure 4 and Figure 5g,l). The dominant fraction of the Ni-rich phase exhibited non-fragmentized lamellae for the alloy 1 with 0.5% Ni after the second step of homogenization (Figure 4a). The partial fragmentation of Ni-bearing particles was observed for the alloy 2 with 2 wt.% Ni (Figure 4b). The fragmentation of the Ni-bearing phase was significant for the alloy 3 with 4% Ni.

**Figure 6:**
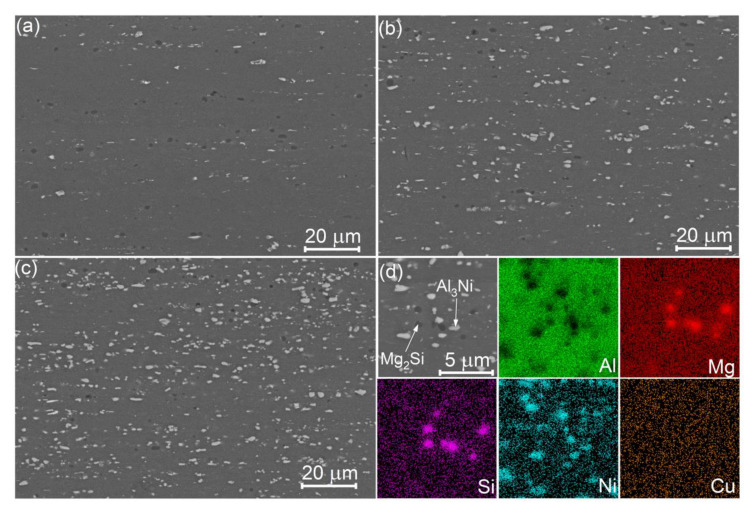
SEM images of cold rolled sheets after final 20 min annealing at 480 °C for the (**a**) alloy 1 (Al-1.2Mg-0.7Si-1.0Cu-0.5Ni), (**b**) alloy 2 (Al-1.2Mg-0.7Si-1.0Cu-2Ni), and (**c**,**d**) alloy 3 (Al-1.2Mg-0.7Si-1.0Cu-4Ni) with corresponded SEM-EDS maps.

Figure 6 shows the SEM micrographs of the alloys studied after a simple thermomechanical treatment and post-deformation annealing at 480 °C for 20 min. A near-homogeneous distribution of the solidification origin particles was observed in the alloys studied after sheet processing.

**Figure 7:**
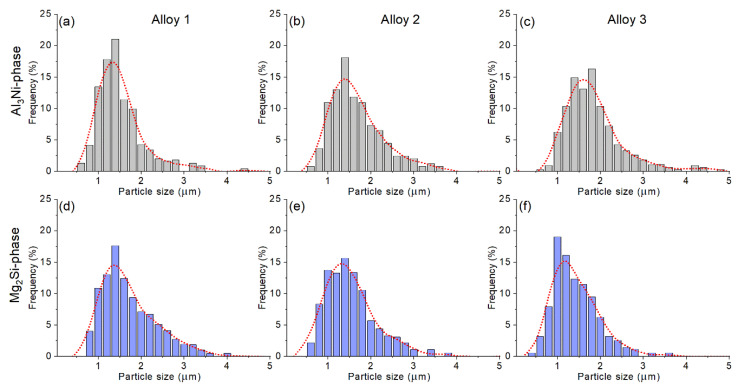
The particle size distribution histograms of the (**a**–**c**) Al_3_Ni and (**d**–**f**) Mg_2_Si phases for the thermomechanical treated (**a**,**d**) alloy 1 (Al-1.2Mg-0.7Si-1.0Cu-0.5Ni), (**b**,**e**) alloy 2 (Al-1.2Mg-0.7Si-1.0Cu-2Ni), and (**c**,**f**) alloy 3 (Al-1.2Mg-0.7Si-1.0Cu-4Ni).

The particles size distribution histograms (Figure 7) exhibited gamma distribution for both types of particles. The mean sizes of bright Ni-bearing particles were 1.7 ± 0.1, 1.6 ± 0.1, and 1.4 ± 0.2, and the mean sizes of Mg_2_Si particles were 1.4 ± 0.1, 1.4 ± 0.1, and 1.6 ± 0.1, for the alloys 1, 2, and 3, respectively.

### 3.4. Dispersoids Parameters

**Figure 8:**
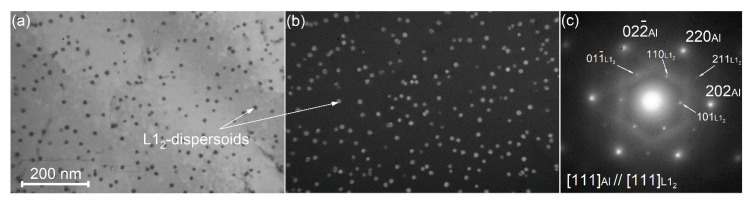
TEM images for the alloy 3 (Al-1.2Mg-0.7Si-1.0Cu-3Ni) after two-step annealing; (**a**) bright field; (**b**) dark field; (**c**) corresponded SAED.

The TEM analysis of the alloy 3 after a two-step annealing revealed a high-density of uniformly distributed nanoscale dispersoids with a mean size of 10 ± 1 nm (Figure 8a,b). The corresponding selected area electron diffraction (SAED) in [111]_l_ zone axis exhibited typical for the L1_2_-phase ordered superlattice reflections (Figure 8c). The [111] zone axis of L1_2_ phase is parallel to [111] zone axis of Al.

### 3.5. Grain Structure for the Thermomechanically Treated Samples

**Figure 9:**
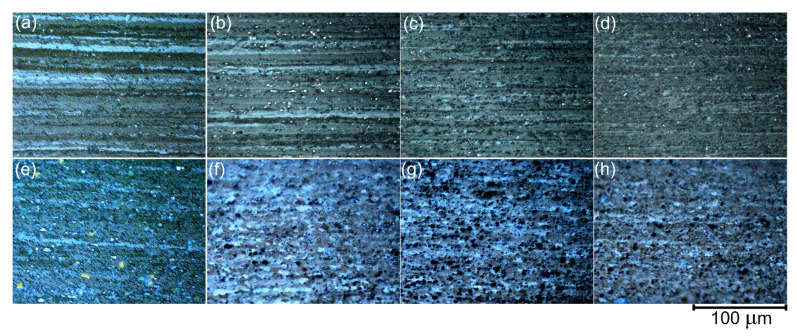
The grain structure for the (**a**,**e**) reference Ni-free alloy, (**b**,**f**) alloy 1 (Al-1.2Mg-0.7Si-1.0Cu-0.5Ni), (**c**,**g**) alloy 2 (Al-1.2Mg-0.7Si-1.0Cu-2Ni), and (**d**,**h**) alloy 3 (Al-1.2Mg-0.7Si-1.0Cu-4Ni) after the thermomechanical treatment and final annealing at (**a**–**d**) 500 °C for 20 min and (**e**–**h**) 300% of strain at 460 °C and constant strain rate of 5 × 10^−3^ s^−1^.

High density of L1_2_ precipitates provided a partially non-recrystallized band-like grain structure after post deformation annealing in a temperature range of 440–500 °C for 20 min (Figure 9). The fraction of the recrystallized grains decreased with increasing volume fraction of Al_3_Ni particles. The mean thickness of non-recrystallized grains decreased with increasing Ni content and were 8.4 ± 1.3, 6.8 ± 0.5, 5.5 ± 0.5 and 4.0 ± 0.3 µm for Ni-free reference alloy, alloy 1, alloy 2, and alloy 3, respectively (Figure 9a–d).

### 3.6. The Tensile Test at Elevated Temperatures

**Figure 10:**
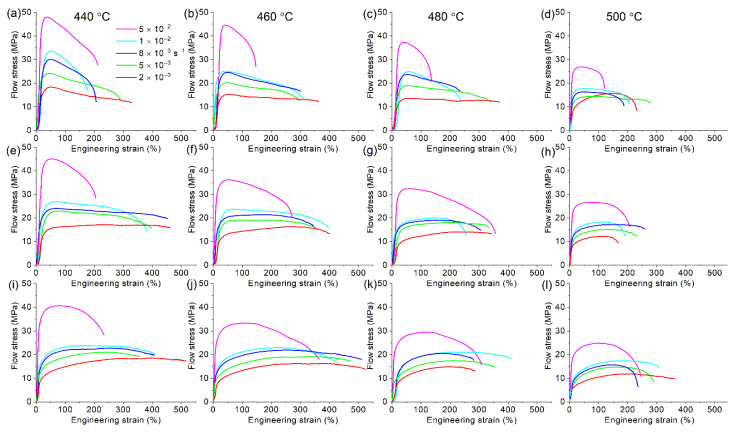
The flow stress-strain dependences for the (**a**–**d**) alloy 1 (Al-1.2Mg-0.7Si-1.0Cu-0.5Ni), (**e**–**h**) alloy 2 (Al-1.2Mg-0.7Si-1.0Cu-2Ni), and (**i**–**l**) alloy 3 (Al-1.2Mg-0.7Si-1.0Cu-4Ni) in a strain rate range of 2 × 10^−3^ s^−1^–5 × 10^−2^ s^−1^ and temperatures of (**a**,**e**,**i**) 440 °C, (**b**,**f**,**j**) 460 °C, (**c**,**g**,**k**) 480 °C, and (**d**,**h**,**l**) 500 °C.

The superplastic deformation behavior was analyzed in a temperature range of 440–500 °C and a constant strain rate range of 2 × 10^−3^–5 × 10^−2^ s^−1^ (Figure 10). The flow stress decreased with increasing temperature and decreasing strain rate. In the studied temperature limits, the maximum stress was observed at the maximum studied strain rate of 5 × 10^−2^ s^−1^. In a temperature range of 440–500 °C, the maximum stress values varied in a range of 27–48 MPa, 26–45 MPa, and 25–40 MPa for alloys with 0.5, 2, and 4 wt.% Ni, respectively. The minimum stress value was achieved at 2 × 10^−3^ s^−1^. In a temperature range of 440–500 °C, the minimum stress values varied in a range of 15–18 MPa, 12–16 MPa, and 10–15 MPa for 0.5, 2, and 4 wt.% Ni, respectively. An increase in Ni content led to a decrease in the flow stress for the studied temperatures.

At the studied deformation conditions, the elongation-to-failure was varied from 130 to 520%. The alloy 1 with 0.5% Ni exhibited superplasticity with elongation-to-failure of 300–370% at 440–480 °C and strain rates of (2–8) × 10^−3^ s^−1^ (Figure 10a–c). At higher strain rates, the elongation did not exceed 200%. The alloys with 2 and 4% Ni exhibited a similar deformation behavior. For the alloy 2 with 2% Ni, the maximum elongation-to-failure of 370–400% was achieved at temperatures of 440–460 °C in a strain rate range of 2 × 10^−3^ s^−1^–1 × 10^−2^ s^−1^ (Figure 10e,f). The maximum elongation of 520% was achieved at 460 °C and (2–8) × 10^−3^ s^−1^ for the alloy 3 with 4% Ni (Figure 10j). At the higher strain rates of (1–5) × 10^−2^ s^−1^, the alloy exhibited elongation of 350–420%. For the decreased temperature of 440 °C, at high strain rates of 5 × 10^−2^ s^−1^, the elongation was below 230%, whereas a strain rate of 2 × 10^−3^ s^−1^ provided the elongation of 520% (Figure 10i). The temperature increase to 480–500 °C resulted in a decrease in elongation in both alloys 2 and 3 (Figure 10g,h,k,l). The elongation of 360% at 500 °C was achieved for the alloy 3 with 4% Ni at a low strain rate of 2 × 10^−3^ s^−1^ (Figure 10l).

**Figure 11:**
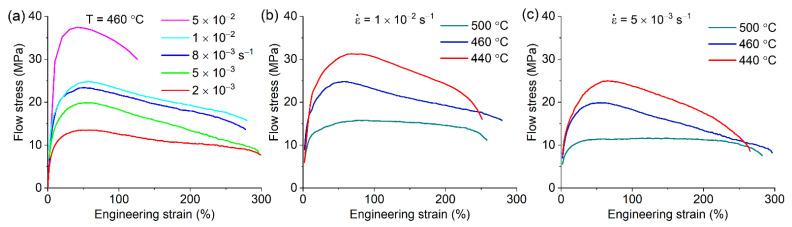
The flow stress-strain dependences for the reference Ni-free alloy at (**a**) 460 °C in a strain rate range of 1 × 10^−2^−5 × 10^−3^ s^−1^ and at constant strain rates of (**b**) 1 × 10^−2^ s^−1^ and (**c**) 5 × 10^−3^ s^−1^ for 440, 460 and 500 °C.

The superplastic behavior of the Ni-free reference alloy was analyzed in similar testing conditions. The alloy exhibited elongation-to-failure of 250–300% in a strain rate range of 2 × 10^−3^–1 × 10^−2^ s^−1^ and a temperature range of 440–500 °C (Figure 11), and non-superplastic flow accompanied by necking with 120% of elongation was observed at a constant strain rate of 5 × 10^−2^ s^−1^.

**Figure 12:**
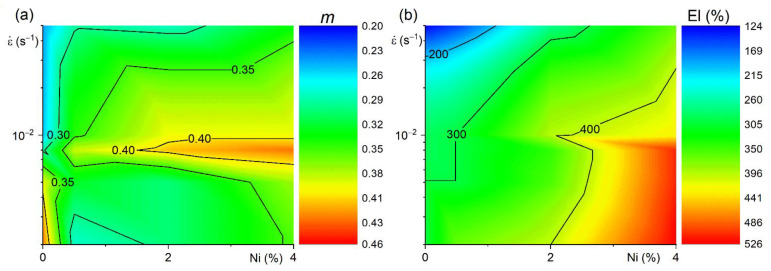
The dependences of (**a**) strain rate sensitivity *m*-coefficient and (**b**) elongation to failure vs. Ni content (x-axis) and strain rate (y-axis) at 460 °C.

The strain rate sensitivity coefficient *m* was determined at 460 °C for 50% strains (Figure 12a). The *m*-values were above 0.3 in a strain rate range of (2–5) × 10^−3^ s^−1^ for the alloy with 0.5%Ni. The *m*-values of 0.35–0.45 were observed in the alloys with 2% and 4% Ni in a strain rate range of 5 × 10^−3^–5 × 10^−2^ s^−1^. For the Ni-free alloy, the coefficient *m* was above 0.3 in a strain rate range of (2–8) × 10^−3^ s^−1^ and below 0.3 at higher strain rates. An *m* coefficient value higher than 0.35 resulted in increased elongation to failure at 460 °C (Figure 12b).

The dynamic recrystallization occurred during the superplastic deformation and provided a fine-grained structure formation for Ni-bearing alloys containing coarse Al_3_Ni particles. After deformation of 300% at 460 °C with a constant strain rate of 5 × 10^−3^ s^−1^, a mean grain size was 9.9 ± 0.6, 7.2 ± 0.6, and 5.9 ± 0.6 µm for the alloys 1, 2, and 3, respectively (Figure 9f–h). The reference alloy that contained a low fraction of coarse particles demonstrated partially non-recrystallized grain structure, even after the failure at 300% and a mean grain size in the recrystallized areas was 16 ± 1 µm (Figure 9e).

## 4. Discussion

The thermodynamic calculation, XRD analysis and SEM studies confirmed the presence of Mg_2_Si phase, and the Al_3_Ni phase of crystallization origin. For an as-cast state, a small amount of non-equilibrium (Si) phase also was found by SEM-EDS. The (Si) phase was dissolved during homogenization annealing. The bright particles of crystallization origin belonged to Al_3_Ni phase were found in as-cast state. The non-equilibrium Al_2_Cu phase observed in as-cast state was dissolved during homogenization. Meanwhile, the SEM-EDS analysis for the bright phase after homogenization revealed an increased concentration of Cu, comparing it to that of for the aluminum-based solid solution (Al). This can be a consequence of partial substitution of Ni atoms by Cu atoms in Al_3_Ni phase or the result of Al_2_Cu phase that was incompletely dissolved during the homogenization. It can also be suggested that Cu-rich Al_7_Cu_4_Ni-phase can be formed in the alloy 1 with low Ni/Cu ratio [67].

The Mg_2_Si phase was fragmentized and spheroidized during homogenization of the studied alloys. The microstructural studies of the as-annealed samples revealed differences in the evolution of the Al_3_Ni lamellae. The fragmentation was observed for the alloys 2 and 3 with increased Ni content, whereas Al_3_Ni phase retained its elongated shape in the alloy 1 with 0.5% Ni. It can be suggested that Cu atoms were dissolved in the Al_3_Ni phase and increased its thermal stability and inhibited fragmentation. The increasing of Ni content results in a higher fraction of Al_3_Ni eutectic phase and, therefore, the effect of Cu becomes increasingly insignificant. After the thermomechanical treatment, a near-uniform distribution of the Al_3_Ni and Mg_2_Si particles with a mean size of 1.4–1.6 µm was observed.

Nickel has an extremely low solubility in aluminum at conventional solidification conditions [68], and the dissolution of Ni in the Sc and Zr bearing precipitates was not observed by atomic probe tomography [69,70], therefore, Ni cannot significantly influence the precipitation kinetics and parameters of dispersoids. It has been suggested that the precipitation behavior of the alloys with different Ni content was similar. It should be mentioned that the applied homogenization annealing provided L1_2_-Al_3_(Sc,Zr) dispersoids with a size of about 10 nm that is similar to the size of precipitates formed at low temperature annealing [43]. We also suggest that Si atoms partially substitute Al in L1_2_ phase providing a more complex dispersoids of (Al,Si)_3_(Sc,Zr) phase as it was demonstrated in Refs. [71,72]. The zone axis [111] of the dispersoids agglomeration was parallel to the corresponding zone axis of the Al matrix that suggested coherency/semi-coherency for the dispersoids and Al matrix. The alloys exhibited partially unrecrystallized grain structure before the start of the superplastic deformation up to a temperature of 500 °C. This emphasizes a strong dislocation pinning effect, due to the L1_2_ dispersoids, which inhibited recrystallization during heating to the superplastic deformation temperature.

The role of the secondary particles on the superplastic behavior is follows. The well-known superplastic deformation mechanisms are grain boundary sliding, dislocation and diffusional creep [10,73,74,75,76,77,78]. Due to the diffusion-controlled nature of these mechanisms, the superplasticity is usually observed at elevated temperatures and low strain rates. Grain refinement simplifies grain boundary sliding and its accommodation by dislocation and diffusional creep, therefore, superplastic behavior is observed at higher strain rates and lower temperatures [20,79]. The bimodal particles size distribution with Al_3_Ni and Mg_2_Si particles of about 1–2 µm in size, and L1_2_ precipitates with size of 10 nm contributed significantly to the grain refinement and provided high strain rate superplasticity for the studied alloys. The Al_3_Ni phase particles accumulate dislocations near interphase boundaries and provide the pronounced PSN effect during the superplastic flow [20,42,58]. Due to PSN mechanism, Al_3_Ni particles increased the dynamic recrystallization rate during the deformation and provided grain refinement. The reference alloy without Ni and Al_3_Ni particles demonstrated weak superplasticity at higher strain rates due to suppressed dynamic recrystallization. In addition, Al_3_Ni particles form interphase boundaries with aluminum matrix. An increased fraction of the interphase boundaries as well as grain boundaries can accelerate the alloy diffusivity and support the acting of diffusion-controlled superplastic deformation mechanisms. As a result, an increase in Al_3_Ni phase fraction increased the elongation-to-failure and decreased the flow stress values. The fine L1_2_ precipitates provided a strong Zener pinning effect, inhibiting dynamic grain growth and providing a stable flow during superplastic deformation.

## 5. Conclusions

The microstructure and the superplastic deformation behavior were studied for the Al-1.2Mg-0.7Si-1Cu-xNi-0.1Sc-0.2Zr alloys, where x = 0, 0.5, 2, and 4 wt.%. The sheets were processed by a simple thermomechanical treatment included homogenization annealing, hot and cold rolling. The main conclusions are drawn as follows:

1. The bimodal particles size distribution with the coarse crystallization-origin particles of the Mg_2_Si and Al_3_Ni phases with 1.4–1.6 µm mean size and L1_2_-structured dispersoids of the Al_3_(Sc,Zr) phase with a mean size of 11 ± 1 nm were formed in the studied alloys. The volume fraction of the Mg_2_Si phase was 1.2% and the volume fraction of the Al_3_Ni phase increased from 2.8 to 8%, with increasing Ni-content from 0.5 to 4%.
2. The alloys studied exhibited superplasticity in a strain rate range of 2 × 10^−3^–5 × 10^−2^ s^−1^ and a temperature range of 440–500 °C. Due to a particle stimulated nucleation effect, an increase in Al_3_Ni phase fraction provided grain refinement during the superplastic deformation, increased the value of elongation-to-failure and decreased flow stress values. An elongation increased from 250–300% for the alloys, with 0–0.5% Ni to 400–500% for the alloy with 4% Ni, which exhibited superplasticity even at a strain rate of 5 × 10^−2^ s^−1^.

## Figures and Tables

**Table 1:** Chemical composition of the studied alloys (wt.%).

Alloy	Mg	Si	Cu	Ni	Sc	Zr	Fe	Al
Reference	1.2	0.7	1.0	0	0.1	0.2	<0.01	balance
1	1.2	0.7	1.0	0.5	0.1	0.2	<0.01	balance
2	1.2	0.7	1.0	2.0	0.1	0.2	<0.01	balance
3	1.2	0.7	1.0	4.0	0.1	0.2	<0.01	balance

## Data Availability

The raw and processed data required to reproduce these results are available by contacting the authors.
